# Supporting Families During End-of-Life Care in Intensive Care Units: A Qualitative Descriptive Study of Nurses’ Experiences in Saudi Arabia

**DOI:** 10.3390/healthcare14182931

**Published:** 2026-09-09

**Authors:** Waleed M. Alshehri, Mohammed Almutairi, Faihan F. Alshaibany, Thurayya Eid, Bader M. Almutairy, Abdulaziz M. Alodhailah

**Affiliations:** 1 Department of Medical-Surgical Nursing, College of Nursing, King Saud University, Riyadh 11451, Saudi Arabia; 2 Department of Nursing Administration and Education, College of Nursing, King Saud University, Riyadh 11451, Saudi Arabia; 3 Department of Community and Psychiatric Mental Health Nursing, College of Nursing, King Saud University, Riyadh 11451, Saudi Arabia

**Keywords:** family support, psychosocial support, critical care nursing, intensive care units, goals of care, palliative care, Saudi Arabia, reflexive thematic analysis

## Abstract

**Background/Objectives:** Supporting families during end-of-life care in intensive care units is clinically, emotionally, and ethically demanding, and nurses’ experiences of this work in Saudi Arabian intensive care settings remain underexplored. This study explored nurses’ experiences of providing psychosocial and emotional support to families during end-of-life care, including their role in treatment-limitation and goals-of-care discussions. **Methods:** A qualitative descriptive study, analyzed using reflexive thematic analysis from a contextualist position, was conducted with 15 intensive care nurses across three healthcare facilities in Riyadh. Ten were Saudi nationals and five internationally recruited; eleven had Arabic as a first language. Semi-structured interviews were conducted in Arabic (*n* = 11) or English (*n* = 4) between December 2025 and February 2026 and coded in the language of collection. **Results:** Three themes were developed: doing relational work inside a system organized for clinical throughput; carrying what the work leaves behind; and standing between, brokering understanding for families. Nurses described having no formal decision-making authority while shaping what families understood and what the clinical team heard. **Conclusions:** Nurses positioned themselves as intermediaries between families and clinical teams. This relational work was shaped by staffing, formal preparation, access to palliative care, and physician recognition of the nursing role. Strengthening these organizational conditions may support more consistent family care.

## 1. Introduction

End-of-life care in intensive care units (ICUs) involves complex clinical, emotional, relational, and ethical considerations. Families may participate in discussions about goals of care, cardiopulmonary resuscitation, limitation or withdrawal of life-sustaining treatment, and transition to comfort-focused care when patients are unable to participate in decisions themselves [1,2]. Such discussions may occur in circumstances characterized by clinical uncertainty, rapid deterioration, and anticipated loss.

Family involvement is an important component of patient- and family-centered intensive care. Family members may require clear and understandable information, opportunities to ask questions, emotional support, and meaningful inclusion in communication with the healthcare team. Their experiences may be shaped by uncertainty about prognosis, limited familiarity with intensive care technologies, differing views about appropriate treatment, and the emotional burden associated with supporting a critically ill relative [1,3]. Structured measures, including the Critical Care Family Needs Inventory and the Family Satisfaction in the Intensive Care Unit survey, have been developed to assess family experiences and needs systematically [4,5].

Nurses are frequently present at the bedside and are often among the healthcare professionals most accessible to families. Their contributions may include recognizing emotional distress, clarifying or reinforcing information provided by the clinical team, preparing families for clinical changes, facilitating communication with physicians, advocating for patient and family concerns, and supporting comfort-focused care [6,7]. Nurses’ capacity to provide such support may be influenced by staffing levels, workload, professional education, access to palliative care resources, and relationships within the interdisciplinary team [7,8]. In addition, nurses’ assessments of physician–family communication have been associated with higher ratings of the quality of dying in the ICU, indicating the potential importance of nursing involvement in the communication environment surrounding end-of-life care [9].

End-of-life care may encompass several related but distinct processes. Goals-of-care discussions concern the overall priorities and purposes of treatment. Decisions to withhold or withdraw life-sustaining treatment concern whether particular interventions should be initiated, continued, or discontinued. Resuscitation decisions concern the use of cardiopulmonary resuscitation in the event of cardiac or respiratory arrest. Family members who participate as surrogate decision-makers may experience substantial emotional and cognitive burden while attempting to represent a patient’s preferences under conditions of uncertainty [10]. Distinguishing these processes is important because participation in communication or support does not necessarily confer formal authority to make or authorize treatment decisions.

In Saudi Arabia, end-of-life care is informed by Islamic bioethical principles and by the applicable national regulatory frameworks. Islamic bioethical scholarship permits the withdrawal of futile treatment when death is considered inevitable while distinguishing this practice from euthanasia, which remains prohibited [11]. The formal framework governing resuscitation and treatment-limitation decisions derives from Fatwa No. 12086, issued in 1988 by the Permanent Committee for Scholarly Research and Iftaʾ, which permits a do-not-resuscitate order where three competent physicians judge resuscitation to be futile [12]. This ruling forms the basis of the national do-not-resuscitate policy issued by the Saudi Health Council in 2017, under which the order is established by three physicians, including the primary consultant, and family members are informed of the decision but do not authorize it [13]. Implementation nevertheless varies between institutions [14,15]. This policy remained the applicable national framework throughout the data-collection period (December 2025 to February 2026); no superseding national policy had been issued at the time of writing, and the 2017 document continues to be described as the operative legal framework for resuscitation and treatment-limitation decisions in Saudi Arabia [15,16]. The full text is publicly available (reference [13]). Family members in Saudi intensive care units therefore occupy a position in which they may be informed of, and emotionally affected by, treatment-limitation decisions in which they hold no formal authorizing role. Within this context, decision authority refers to the formal authority to authorize a treatment-limitation decision. This is distinct from participation, consultation, communication, advocacy, and emotional support. Nurses may contribute substantially to these latter activities without holding formal authority to authorize treatment-limitation decisions.

Previous research has examined end-of-life experiences among Muslim families and healthcare professionals in Saudi Arabia and other Middle Eastern settings [17,18,19]. However, less is known about how ICU nurses in Saudi Arabia experience and enact their role in supporting families during end-of-life care. In particular, limited attention has been given to how nurses work between families and clinical teams when they do not hold formal decision-making authority but remain closely involved in communication, emotional support, advocacy, and the interpretation of families’ concerns for other members of the healthcare team. Existing Saudi research has examined nurses’ emotional experiences and resilience in palliative and end-of-life settings [20], while international research has identified organizational and interdisciplinary barriers to high-quality end-of-life care [6,7]. Nevertheless, the day-to-day intermediary role of ICU nurses in supporting families, and the organizational and professional conditions that shape this role remain insufficiently characterized.

Accordingly, this study explored nurses’ experiences of providing psychosocial and emotional support to families during end-of-life care in ICUs in Saudi Arabia. This study also examined how nurses described their role in relation to treatment-limitation and goals-of-care discussions while distinguishing their participation in communication, advocacy, and family support from formal decision-making authority.

## 2. Materials and Methods

### 2.1. Ethical Considerations

This study was conducted in accordance with the Declaration of Helsinki and was approved by the Institutional Review Board of King Saud University, Saudi Arabia (KSU-HE-25-1440; 2 December 2025). Administrative and research approvals were obtained from the participating healthcare facilities before recruitment and data collection commenced.

Written informed consent was obtained from all participants before the interviews. The consent process covered participation in the interview, audio-recording, verbatim transcription, translation of Arabic-language transcripts into English, and the use of anonymized verbatim quotations in publications. Participants were informed about the purpose and procedures of this study, the voluntary nature of participation, confidentiality and data-protection measures, and their right to withdraw from this study at any time without consequences. Consent materials were provided in both Arabic and English.

Participants were assigned identification codes, and personally identifying information was removed from transcripts and other analytic materials. Audio recordings and other research data were stored securely on a password-protected institutional server, with access restricted to authorized members of the research team. Digital research data will be retained securely as policy after completion of this study, after which audio recordings and identifiable information will be permanently deleted. The retention period was specified in the protocol approved by the King Saud University Institutional Review Board (KSU-HE-25-1440).

### 2.2. Study Design and Analytic Position

A qualitative descriptive design was used to explore ICU nurses’ experiences of supporting families during end-of-life care [21,22]. Qualitative description aims to provide a comprehensive, data-near account of participants’ experiences while minimizing reliance on an overarching interpretive or theoretical framework.

Data were analyzed using Braun and Clarke’s reflexive thematic analysis [23,24]. Because qualitative description emphasizes a relatively data-near account, whereas reflexive thematic analysis recognizes themes as developed through researchers’ active engagement with the data, the analytic approach was explicitly positioned to maintain descriptive fidelity while acknowledging the interpretive role of the research team. Accordingly, participants’ language, accounts, and clinical referents were retained as closely as possible in reporting, while theme development was understood as an interpretive process undertaken by the research team.

The analysis adopted a contextualist position. Participants’ accounts were treated as accounts of real experiences and institutional conditions while recognizing that these accounts were generated within specific interview contexts. Interviews were conducted in two languages and involved hospital-employed nurses speaking with nurse academics based at the College of Nursing, King Saud University. Interviewers and participants therefore belonged to the same national nursing and higher-education community, but not to the same employing organization: neither interviewer held a clinical, academic, supervisory, or employment appointment at any of the three participating hospitals, and neither had a prior professional or personal relationship with any participant (Section 2.4 and Section 2.5). Shared membership of a national professional community may nevertheless have influenced how experiences were expressed and interpreted, and this is considered further in Section 4.2.

Coding was conducted primarily at the semantic level, with latent interpretation used selectively where patterns across participants’ accounts indicated meanings that were not expressed explicitly. In particular, the intermediary positioning of nurses represented in Theme 3 was developed through interpretation of patterns across accounts rather than treated as a concept directly named by participants. The analytic orientation was experiential, focusing on how nurses described and made sense of their experiences of supporting families during end-of-life care.

A hybrid inductive–deductive coding strategy was used. Inductive codes were developed from participants’ accounts, while a limited set of deductive codes was informed by the domains addressed in the semi-structured interview guide. The research team remained attentive to the possibility that deductive domains could constrain attention to data outside the interview framework. During analysis, codes and candidate themes were reviewed against the transcripts to identify experiences that extended beyond the predefined interview domains and to avoid treating interview-guide categories as predetermined themes.

This approach allowed this study to retain the descriptive emphasis of qualitative description while using reflexive thematic analysis to develop patterns of meaning across participants’ accounts. The researchers’ interpretive role was therefore acknowledged rather than presented as separate from the process of theme development.

### 2.3. Study Setting

This study was conducted between 10 December 2025 and 28 February 2026 in three healthcare facilities in Riyadh, Saudi Arabia: two tertiary teaching hospitals (Site A and Site B) and one specialized private medical center (Site C). All three provide adult intensive care services to Saudi and expatriate populations. The three sites were purposively selected to provide variation in institutional context, including public teaching and private-sector care, and to capture nurses’ experiences across different organizational arrangements for adult intensive care and end-of-life care. Non-identifying characteristics of each participating site are reported in Table 1. Site C did not operate a specialist palliative care service. Complex end-of-life cases at this site were managed by the attending intensivist together with the bedside nursing team, with clinical ethics consultation and spiritual care available on referral or request, and with referral to a palliative care service at another facility where specialist input was required. Nurses at Site C therefore relied on the general ICU team and on individual professional contacts rather than on an in-house specialist service, as reflected in participants’ accounts in Section 3.3.2.

### 2.4. Participants and Recruitment

Purposive sampling was used to recruit nurses with direct experience of supporting families during end-of-life care in the ICU. Eligible participants held valid nursing licensure in Saudi Arabia; had at least two years of ICU nursing experience; had supported families during end-of-life care within the preceding 12 months; were fluent in Arabic or English; and were able to provide informed consent. Nurses in exclusively administrative or non-clinical roles were excluded.

Recruitment information was distributed through three routes: invitation via ICU nurse managers, posted announcements in staff areas, and snowball referral from early participants. Twenty-one nurses were approached in total: five through nurse-manager distribution, ten through staff-area announcements, and six through snowball referral. Four declined participation because of work-schedule constraints, and two who expressed interest did not proceed to an interview because a mutually suitable interview time could not be arranged. The final sample comprised 15 nurses. The recruitment and selection process is summarized in Table 2.

Potential participants contacted the research team directly by email or telephone after receiving the study information. Expressions of interest and consent decisions were therefore communicated directly to the research team rather than to nurse managers. Nurse managers were informed that recruitment materials would be distributed within their units but were not informed of individual participation decisions. Participation was voluntary, and nurses were informed that choosing not to participate or withdrawing would have no effect on their employment or professional evaluation. The research team held no supervisory, educational, employment, or personal relationship with participants.

The study protocol anticipated that participation could evoke emotional discomfort because nurses were asked to discuss end-of-life experiences. However, emotional distress was not operationally applied as an eligibility or exclusion criterion, and no nurse was excluded from participation on the basis of anticipated or observed emotional distress. Participants were informed before the interview that they could decline to answer any question, pause or stop the interview, or withdraw without providing a reason. No distress-based exclusion decision was recorded or used in participant selection.

#### Sample Adequacy

No formal power calculation or predetermined saturation stopping rule was used. Consistent with reflexive thematic analysis, we did not treat data saturation as a stopping rule, since themes are understood as constructed rather than as finite entities awaiting discovery [25]. Sample adequacy was instead assessed using the concept of information power [26]: the study aim was narrow; the participant group was specific (ICU nurses with recent, direct experience of supporting families during end-of-life care); interviews were rich and sustained; the sample spanned three institutions, three ICU subspecialties, and a wide range of experience; and the analysis was descriptive and pattern-based across the dataset rather than theory-generating. We do not claim a formal cross-site comparative analysis: the three sites provided variation in organizational context rather than units of systematic comparison, and site-level comparisons are accordingly not presented in the Results (Section 3.5.5).

Recruitment and analysis proceeded concurrently, and the research team reviewed the developing dataset after every third interview (*n* = 3, 6, 9, 12, and 15). The final three interviews did not introduce a substantially different organizing concept, but they added further examples of the tension between competing clinical demands and family support, and strengthened the developing interpretation of nurses as intermediaries between families and the clinical team. These interviews also contributed additional variation in how nurses described managing emotional demands and communicating information to families. Recruitment was concluded after 15 interviews because the dataset provided sufficient information to address the study aim within the defined population, setting, and methodological approach.

### 2.5. Data Collection

Semi-structured individual interviews were conducted by two nurse researchers who are co-authors of this manuscript: W.M.A., a male nurse researcher, who conducted eight interviews (53.3%), and T.E., a female nurse researcher, who conducted seven interviews (46.7%). Each interview was conducted in Arabic or English according to the participant’s preferred language.

Both interviewers held PhDs in Nursing and faculty appointments at the College of Nursing, King Saud University. They had clinical backgrounds in critical care nursing and formal training and experience in qualitative research. Neither interviewer held a clinical, supervisory, educational, or employment appointment at the participating hospitals or ICUs, and neither had a prior professional or personal relationship with any participant. Their institutional affiliation was to the College of Nursing, King Saud University, which is organizationally separate from the three participating healthcare facilities (Section 2.2). This separation was maintained throughout recruitment, interviewing, and analysis to minimize potential power differentials and perceived pressure to participate.

Before each interview, participants were informed that the interviewer was a nurse researcher conducting this study to understand nurses’ experiences of supporting families during end-of-life care in the ICU. Participants were explicitly informed that the interview was conducted for research purposes only and was not a clinical assessment, performance evaluation, or employment-related activity. They were reminded that participation was voluntary, that they could decline to answer any question, and that they could pause or withdraw from the interview at any time without consequence.

Interviews were conducted individually in private locations selected by participants, typically quiet hospital rooms or offices, with no third party present. Interviews lasted 35–50 min, with a mean duration of 42 min (SD 4.6). With participants’ permission, all interviews were audio-recorded and transcribed verbatim by members of the research team. Brief contextual field notes were made by the interviewer immediately after each interview and were used to support interpretation during analysis. Transcript accuracy was verified by the first author through comparison of the transcripts with the original audio recordings. No repeat interviews were conducted, and transcripts were not returned to participants for correction or comment.

#### 2.5.1. Interview Guide Development

The semi-structured interview guide was developed following a review of relevant literature on family support in critical care and end-of-life communication and consultation with clinical experts in ICU nursing and end-of-life care. The guide explored nurses’ experiences of supporting families during ICU end-of-life care, communication strategies, factors perceived to facilitate or hinder family support, nurses’ emotional responses, interactions with families and other healthcare professionals, institutional influences, and recommendations for improvement.

The interview guide was pilot-tested with three ICU nurses who met the eligibility criteria but were not included in the final sample. Based on pilot feedback, minor revisions were made to the wording and sequencing of questions and to the clarification of selected prompts. During the interviews, open-ended questions and follow-up probes, such as “Can you tell me more about that?” and “What happened then?”, were used to encourage detailed and contextualized accounts. The final interview guide is provided in Supplementary File S1.

#### 2.5.2. Language, Translation, and Analytic Language

Eleven interviews were conducted in Arabic and four in English. Both interviewers were fluent in Arabic and English and conducted interviews according to the participants’ preferred language.

Initial coding was conducted in the original language of each interview, allowing participants’ wording, meanings, and culturally specific expressions to be considered during the analytic process. Arabic-language data were coded in Arabic by Arabic-speaking members of the research team, while English-language interviews were coded in English. Translation into English was undertaken only for quotations selected for presentation in this manuscript, and therefore occurred after coding and theme development rather than before.

Where interviews were conducted in Arabic, quotations selected for presentation were translated into English while preserving the meaning and intended expression of participants’ accounts. Forward translation was undertaken by A.M.A., a bilingual member of the research team with qualitative research experience, and independent back-translation into Arabic was undertaken by F.F.A., a second bilingual nurse researcher who had not participated in coding the transcript concerned, following established procedure for cross-cultural translation [27]. The first author compared the original Arabic quotation, the English translation, and the back-translated version to confirm that the intended meaning and contextual nuance had been retained, and any discrepancies were resolved through discussion between the bilingual researchers.

Quotations were minimally edited for clarity and readability, including removal of filler words and potentially identifying information and minor grammatical adjustments where necessary, without altering their substantive meaning or emotional tone. Where culturally specific Arabic expressions could not be adequately conveyed through direct translation, the original expression was retained where appropriate and accompanied by a brief contextual explanation, because a literal rendering would have reduced a culturally situated meaning. All presented quotations were checked against the source transcripts and audio recordings, and each translation was confirmed to reflect the participant’s account accurately.

The research team considered the linguistic context of participants’ accounts throughout analysis. Differences in language were not treated as evidence of substantive group differences unless supported by the data. Instead, language was considered when interpreting meaning, context, and culturally specific expressions.

### 2.6. Data Analysis

Data were managed using NVivo 12 (QSR International, Melbourne, Australia) and analyzed using Braun and Clarke’s six-phase approach to reflexive thematic analysis [23,24]. The analysis was iterative and reflexive rather than oriented toward achieving coding consensus or inter-coder reliability. The six phases, including familiarization with the data, generating initial codes, constructing candidate themes, reviewing themes, defining and naming themes, and producing the report, were treated as recursive and overlapping processes.

The first author (W.M.A.) read and re-read all 15 interview transcripts in their original language before and during coding, with approximately 20 h of direct engagement with the transcripts before completion of the initial coding cycle. Initial coding was conducted line-by-line, with attention to participants’ experiences of supporting families during end-of-life care, communication practices, emotional experiences, contextual influences, and perceived facilitators and barriers.

A second researcher (M.A.) independently coded six of the 15 transcripts (40%). The transcripts were selected to provide variation across the interview sequence, participating institutions, ICU contexts, and interview languages. This additional coding was undertaken as a reflexive analytic resource rather than to calculate inter-coder reliability. No inter-rater reliability statistics, percentage agreement, or formal consensus coding procedures were used. Instead, the second researcher’s interpretations were used to surface alternative readings, question taken-for-granted assumptions, and identify areas requiring further engagement with the underlying data.

The researchers met approximately every two to three interviews for 30–45 min to discuss emerging interpretations and divergent readings. Differences in interpretation were not automatically resolved by selecting a single correct code. Rather, they prompted the researchers to return to the relevant transcript segments, consider the broader context of participants’ accounts, and examine how their professional and interpretive positions influenced the analysis. Analytic decisions, revisions to codes, and development of themes were documented through NVivo analytic memos and a shared reflexive record.

Coding was undertaken using a flexible coding framework rather than a fixed a priori codebook. Codes were generated in relation to participants’ accounts and were iteratively revised, merged, expanded, renamed, or discarded as the analysis progressed. Candidate themes were developed by grouping conceptually related codes and examining patterns of shared meaning across participants. Candidate themes were reviewed against supporting extracts and subsequently against the full dataset to assess internal coherence, distinctiveness, and relevance to the research question. Themes were then refined, defined, and named to capture the central organizing concept of each pattern of meaning.

The first author retained responsibility for the final analytic interpretation following iterative discussion with the second researcher. The second researcher’s contribution was therefore considered a critical and reflexive perspective rather than a reliability check. The final thematic structure reflected the first author’s interpretation informed by discussion, alternative readings, repeated engagement with the dataset, and consideration of competing explanations.

One documented divergence concerned participants’ descriptions of family involvement in end-of-life decision-making. While several accounts initially suggested that family involvement was experienced primarily as supportive and necessary, closer examination identified accounts in which extensive family involvement was experienced by nurses as emotionally demanding and, at times, as complicating communication. Rather than treating these accounts as contradictory data to be excluded, the researchers revisited the relevant transcripts and refined the interpretation to recognize family involvement as context-dependent, involving both supportive and challenging dimensions.

### 2.7. Reflexivity and Trustworthiness

The research team comprised PhD-prepared nurses with academic appointments at the College of Nursing, King Saud University, clinical experience in critical care, and prior training and experience in qualitative research. The team included both male and female researchers. The researchers’ professional and institutional positions were considered throughout this study because these positions could influence what was noticed, emphasized, questioned, or interpreted during data collection and analysis.

The researchers were nurse academics working within the broader Saudi healthcare and educational system in which participants were professionally situated. Their clinical and academic backgrounds and commitment to supporting families during serious illness and end-of-life care increased their sensitivity to relational care, communication, and organizational conditions affecting nurses’ interactions with families. At the same time, this professional positioning could make certain interpretations more readily available, particularly interpretations that framed difficulties as deficiencies in communication training or organizational support. The researchers therefore remained attentive to alternative explanations, including emotional demands, family preferences, workload pressures, ethical tensions, and contextual constraints.

Reflexivity was incorporated through reflexive journaling, analytic memos, and regular research-team discussions. After several early interviews, the first author noted an initial tendency to interpret nurses’ accounts of difficult family interactions primarily as communication challenges. Subsequent analysis deliberately examined whether these accounts also reflected emotional burden, competing clinical priorities, family expectations, and ethical tensions. This reflexive process resulted in greater attention to the interaction between communication practices and the emotional and organizational context in which family support occurred.

Trustworthiness was supported through repeated engagement with the complete dataset, reflexive documentation, peer debriefing, maintenance of an audit trail, and examination of divergent accounts. Peer debriefing was conducted between the first and second researchers approximately every two to three interviews, with discussions lasting approximately 30–45 min. These discussions focused on emerging codes, assumptions, alternative interpretations, and the development and refinement of candidate themes. The audit trail consisted of NVivo analytic memos, coding revisions, thematic notes, and documented decisions regarding the interpretation and refinement of themes. Taken together, these procedures address credibility, dependability, and confirmability as set out for naturalistic inquiry [28].

Participant feedback was also incorporated as a dialogic component of the analytic process. Five participants were purposively selected to provide variation in clinical experience and participating facility. Each received a two-page summary describing the developing themes and was asked whether the summary reflected their experiences and whether any important perspectives were missing. Feedback was obtained electronically, and four of the five participants responded. Participants generally considered the themes recognizable and relevant, while two suggested that the emotional impact of supporting families was particularly important. This feedback prompted the researchers to further examine emotional experiences across the dataset and strengthen the representation of emotional demands within the relevant theme. Participant feedback was treated as an opportunity to assess interpretive resonance rather than as objective validation or correction of the researchers’ analysis.

Divergent cases were deliberately examined during theme development. Accounts that did not readily fit emerging patterns were retained and revisited in their original context. For example, although many participants described family involvement as an important component of high-quality end-of-life care, some participants described situations in which family involvement increased communication difficulties or emotional burden. These accounts were not treated as exceptions to be removed; instead, they contributed to a more differentiated interpretation in which family involvement was understood as potentially supportive while also generating emotional and ethical challenges depending on the clinical context.

This study was conducted between 10 December 2025 and 28 February 2026. This period represents the data-collection period and was not characterized as prolonged engagement. Trustworthiness was considered in relation to transparency of the analytic process, reflexive engagement, interpretive coherence, consideration of alternative accounts, and traceability of analytic decisions rather than through claims of objective reliability.

This manuscript was reviewed against the 32-item Consolidated Criteria for Reporting Qualitative Research (COREQ) checklist [29], which is provided as Supplementary File S2, with section references identifying where each reporting item is addressed. Data saturation is not claimed.

## 3. Results

### 3.1. Participant Characteristics

Fifteen nurses participated. Participants ranged in age from 28 to 58 years (mean 41.0, SD 8.2). Twelve were female. Participants had between 2 and 32 years of nursing experience and between 2 and 25 years of ICU experience. Participant characteristics are presented in Table 3.

### 3.2. Overview of Themes

**Figure 1:**
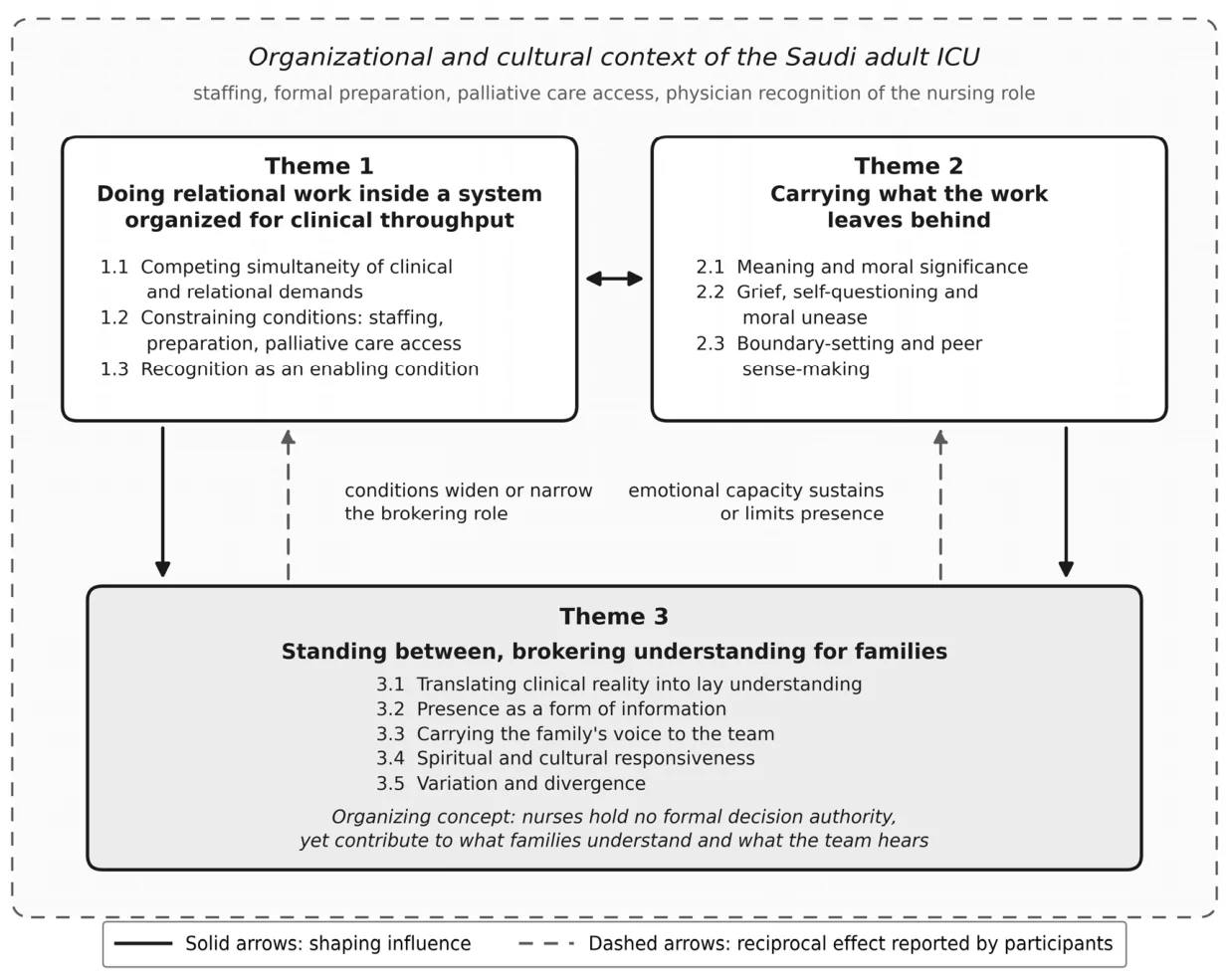
Thematic map showing the relationships between the three themes and their subthemes. Solid arrows indicate shaping influence; dashed arrows indicate reciprocal effects reported by participants.

Three themes were developed. Their relationships are shown in Figure 1. Themes 1 and 2 describe the conditions, organizational and emotional, respectively, within which nurses worked. Theme 3 describes what nurses did within those conditions and carries this study’s central analytic claim.

### 3.3. Theme 1: Doing Relational Work Inside a System Organized for Clinical Throughput

#### 3.3.1. Competing Simultaneity of Clinical and Relational Demands

Participants described family support not as a task that followed clinical work but as one that ran concurrently with it, and the difficulty they described was simultaneity rather than volume. Monitoring, medication administration, documentation, and the needs of other critically ill patients did not pause while a relative was distressed.

“Some days, honestly, you feel pulled in every direction. You are monitoring the ventilator, giving medications, doing your documentation, and then you see a family member crying. You want to sit with them, talk to them, help them understand what’s happening, but you have other patients, and then an alarm is going off in the next bed. You do what you can and then you have to move on. But sometimes you leave thinking, ‘I should have done more.’”

(Participant 1)

Participants triaged their attention by patient acuity and family distress, and described the residue of that triage as a sense of insufficiency rather than of failure.

“When a patient is actively dying, the family needs someone to explain what’s happening, answer their questions, and sometimes just sit with them. But I cannot leave my other patients. If my other patients are stable, I try to stay a little longer. If they’re not, I have to go.”

(Participant 2)

#### 3.3.2. Constraining Conditions

Participants described staffing pressures, limited formal preparation, and restricted access to palliative care as conditions that narrowed what they could do for families. Rather than describing these limitations as a lack of willingness or ability, participants located the difficulty in how nursing time and support were organized within the ICU.

“When one patient is dying, the family needs a lot of support. But I still have other patients to care for. You can’t just stop everything else. The system doesn’t always recognize how much time it takes to sit with a family and support them, especially emotionally.”

(Participant 6)

Another participant described the same tension between family needs and competing clinical responsibilities:

“Sometimes you want to stay with the family because you can see they really need you, but then you have another patient calling you or an alarm going off. You have to leave, even when you feel that the family still needs you.”

(Participant 2)

These accounts suggest that staffing constraints affected not only the amount of time available for family support but also nurses’ ability to remain emotionally present during particularly difficult moments.

Participants also described limited preparation for end-of-life communication. Thirteen of the 15 participants (86.7%) reported no formal educational preparation in end-of-life care, and only two had received structured preparation (Table 3). Participants described learning instead through clinical exposure, observation, and guidance from colleagues.

“Nobody really taught me how to have these conversations. The first time I sat with a family to discuss withdrawal of ventilatory support, I was nervous. I didn’t know what to say, and I was afraid I would say the wrong thing. I learned gradually from colleagues and just from being in these situations and doing it.”

(Participant 7)

Participant 7 described feeling uncertain when first participating in conversations with families concerning withdrawal of ventilatory support. In this context, the nurse described being present to support the family, to clarify information already communicated by the clinical team, and to respond to questions, rather than independently making or authorizing the treatment decision.

A second participant similarly described learning through experience rather than structured preparation:

“You learn as you go. There was no specific training where someone sat down with us and explained how to talk to a family when the patient is dying. You watch other nurses, you listen to how they talk, and then you slowly become more comfortable.”

(Participant 13)

Restricted access to specialist palliative care created a further constraint. In the absence of readily available specialist support, nurses described having to improvise or rely on informal professional networks when families required support beyond their own expertise.

“I work in a hospital without a palliative care team. When families need that specialized support, I have to try to find someone I know at another hospital. It’s not easy, and sometimes I feel like I’m being asked to provide something that I’m not really trained to provide.”

(Participant 8)

Another participant described the difficulty of managing complex family needs without specialist input:

“There are situations where you feel the family needs more than what you can give them. You want someone from palliative care to come and help, but if that service is not available, you are left trying to manage the situation yourself.”

(Participant 15)

Together, these accounts show that the constraints were not limited to workload alone. They also involved gaps in preparation and restricted access to specialist palliative care resources, which shaped how much time, confidence, and support nurses could bring to family care.

#### 3.3.3. Recognition as an Enabling Condition

Enabling conditions were described less frequently and in less detail than constraining conditions. Rather than presenting the two as equally prominent, the analysis retains this asymmetry. Where participants identified conditions that made family support more possible, these primarily concerned recognition of nurses’ bedside knowledge and their contributions to understanding family concerns. Rather than describing extensive formal resources, participants emphasized the importance of being listened to and having their observations taken seriously by other members of the clinical team.

“The doctors listen to us, and that really helps. We spend a lot of time with patients and families, and we notice things that they may not see. When they value what we tell them about the family’s concerns and needs, it makes a big difference.”

(Participant 11)

Another participant emphasized the practical effect of being listened to:

“When the doctor asks what I think the family understands or what they are worried about, it helps a lot. I feel that I can explain what I have seen because I’m with the patient and family for most of the shift.”

(Participant 9)

Participants also described supportive team relationships as making it easier to respond to families:

“If the team is working together and everyone is communicating, it makes things much easier. You know who to ask, you know what information has been given to the family, and you don’t feel like you’re handling everything alone.”

(Participant 4)

These accounts suggest that recognition, communication, and collegial support could make family support more possible within existing organizational constraints. The enabling condition was therefore relational rather than primarily resource-based: nurses described being better able to contribute when their knowledge of the patient and family was acknowledged by the wider team.

### 3.4. Theme 2: Carrying What the Work Leaves Behind

#### 3.4.1. Meaning and Moral Significance

Participants described end-of-life care as difficult but also as some of the most meaningful work they performed. The significance they attached to this work was expressed through being present for families, helping them understand what was happening, and supporting them at the end of a patient’s life.

“It’s hard, but it’s also something important. When you help a family understand what’s happening, or you are there when they’re saying goodbye, you feel like you are part of something that matters. It’s heavy, yes, but it means something.”

(Participant 3)

Another participant described meaning in being able to provide comfort even when the clinical situation could not be changed:

“You may not be able to change what is happening to the patient, but you can still do something for the family. Sometimes just explaining things or staying with them for a few minutes makes you feel that you have done something important.”

(Participant 12)

A further participant linked meaningfulness to being present during the family’s final moments:

“When the family is saying goodbye, you know that moment is very important for them. Even if you are just there and helping them, you feel that your presence has a purpose.”

(Participant 14)

For these participants, meaning did not remove the emotional difficulty of the work. Instead, the two experiences existed together. The same encounters that were emotionally demanding could also give nurses a sense that their work had purpose.

#### 3.4.2. Grief, Self-Questioning, and Moral Unease

Participants described grief, retrospective self-questioning about their own conduct, and unease when they perceived care as non-beneficial. These experiences were described as part of the emotional and moral complexity of end-of-life nursing rather than as clinical diagnoses or measured psychological states. They are reported in participants’ own terms rather than classified using constructs such as burnout, compassion fatigue, moral distress, or resilience, none of which was assessed in this study.

“Sometimes after I go home, I keep thinking about the family. I think, ‘Did I do enough for them? Did I spend enough time with them?’ You know you were busy, but you still wonder if you could have done more.”

(Participant 1)

Another participant described the difficulty of carrying a particular encounter after the shift had ended:

“Sometimes you leave the ICU and the situation stays in your mind. You remember the patient and the family, and you start thinking about what happened and whether there was something else you could have done.”

(Participant 5)

Participants also described unease when continuing treatment appeared inconsistent with what they understood to be beneficial for the patient:

“Sometimes you look at the patient and you feel that there is not much more that can be done, but the treatment continues. That is difficult because you are thinking about the patient and the family, and you are not sure what is really helping anymore.”

(Participant 10)

These accounts suggest that the emotional burden of end-of-life care was not confined to the clinical encounter itself. Nurses sometimes revisited these situations afterwards, questioning their own actions or feeling uneasy about the direction of care.

#### 3.4.3. Boundary-Setting and Peer Sense-Making

Participants described learning to create boundaries between their work and their lives outside the ICU. They also described talking with colleagues as an important way of processing difficult experiences. These practices were presented as skills developed through experience rather than as fixed personal characteristics.

“I’ve learned that I can’t take everyone’s grief home with me. I do my best while I’m with the family, and then when my shift is finished, I try to leave it there. Talking with colleagues helps because they understand. They’ve seen these situations too.”

(Participant 4)

Another participant described deliberately creating a separation after difficult shifts:

“At the beginning, I used to think about the patients a lot when I went home. Now I try to tell myself, ‘You did what you could during your shift.’ It doesn’t mean you don’t care. You just have to learn how to leave some of it at work.”

(Participant 7)

Colleagues were also described as an important source of shared understanding:

“Sometimes you just need to talk to another nurse who was there. You don’t have to explain why it was difficult because they already understand. We talk about what happened, and sometimes that helps you look at it differently.”

(Participant 13)

For some participants, peer discussion was also a way of checking their own responses to difficult situations:

“When something stays in my mind, I usually talk to one of my colleagues. We might say, ‘What do you think? Could we have done something differently?’ It helps because sometimes you are looking at the situation from only one side.”

(Participant 11)

Taken together, these accounts suggest that nurses managed the emotional residue of end-of-life care through a combination of personal boundary-setting and shared sense-making with colleagues. These practices did not eliminate the emotional weight of the work, but they helped nurses create some separation between their experiences in the ICU and their lives beyond it.

### 3.5. Theme 3: Standing Between, Brokering Understanding for Families

Across accounts, participants described a position rather than a set of tasks: standing between a family and a clinical team, holding no authority over what was decided while contributing to what the family understood and what the team heard. This intermediary positioning is the organizing concept of the theme, and it is what makes translating information, remaining present, and carrying family concerns upward a single phenomenon rather than a list of desirable activities. Participants did not name this positioning; it was constructed from the pattern across accounts, and Section 2.2 states the latent level of interpretation involved.

#### 3.5.1. Translating Clinical Reality into Lay Understanding

Participants described converting clinical information into language families could act on, and described checking comprehension as part of the translation rather than as a courtesy.

“I try to use simple words, not too much medical language. Instead of saying that the patient has multiorgan failure, I might say that the body is starting to shut down. Then I pause and ask, ‘Do you understand?’ or ‘Do you have any questions?’”

(Participant 12)

#### 3.5.2. Presence as a Form of Information

Participants described silent presence as communicating something that words did not, and distinguished it from the absence of information rather than treating it as a substitute for explanation.

“Sometimes families don’t need more information. They need to know they’re not alone. I may sit quietly with them, let them cry, or hold their hand. You don’t always have to say something. Sometimes just being there is enough.”

(Participant 14)

#### 3.5.3. Carrying the Family’s Voice to the Team

Participants described their role as helping the clinical team understand what mattered to the patient and family. Rather than seeing themselves as decision-makers, nurses described listening to family concerns, clarifying what the family understood, and bringing their questions and preferences back to the wider team. This positioned advocacy as a form of brokering between the family and clinicians.

“I ask families what their loved one would have wanted. They may not remember the exact words, but they often remember what was important to the patient. Then we try to connect that with what is happening now.”

(Participant 12)

Another participant described the importance of making sure that family concerns were not lost during discussions with the clinical team:

“I see myself as an advocate for the family. When they tell us what they want or what they’re worried about, I make sure the team hears it. If there is disagreement, I try to explain where the family is coming from and help both sides understand each other.”

(Participant 10)

Participants generally located their influence in what they communicated to the team rather than in the final treatment decision. One nurse explained:

“I can tell the doctor what the family is saying and what they are asking for, but the final decision is not mine. My role is to make sure their concerns are not missed.”

(Participant 8)

#### 3.5.4. Spiritual and Cultural Responsiveness

Participants described adapting family support to individual spiritual and cultural preferences rather than assuming that all families wanted the same forms of support. This included asking about preferences for family presence, prayer, communication, and access to spiritual support where these needs were raised by families.

“When the family wants to pray, we try to give them the space and time to do that. At that point, you understand that it is not only about the medical care. They need to be with their loved one and have that spiritual time.”

(Participant 6)

A participant also emphasized that families varied in how they wanted information and support:

“Every family is different. Some want you to explain everything in detail, and some just want to stay close to the patient. I try to ask them what they need rather than assuming that I already know.”

(Participant 14)

Family presence was similarly described as something nurses tried to accommodate within institutional requirements:

“When the patient is getting close to death, the family wants to be there. Sometimes there are many family members, and we have to work with the ICU rules, but if we can make it possible, we try to give them that time together.”

(Participant 9)

These accounts positioned cultural and spiritual responsiveness as part of nurses’ relational work rather than as a separate clinical task. Nurses described negotiating institutional policies while attempting to preserve forms of family presence, spiritual practice, and communication that were meaningful to individual patients and families.

#### 3.5.5. Variation and Divergence

Participants did not describe the brokering role in exactly the same way. For some nurses, the role was primarily informational, involving clarification of medical explanations and communication of family questions to the clinical team:

“Usually, they just want someone to explain what the doctor said. I listen first, then I make sure they understand, and if they still have questions, I take those questions back to the team.”

(Participant 5)

For others, brokering involved more active mediation when families and clinicians appeared to understand the situation differently:

“Sometimes the family understands things one way and the medical team is looking at it another way. Then I try to explain to the family what the team means, and at the same time I tell the team what the family is really worried about.”

(Participant 10)

Participants did not describe the brokering role as uniformly experienced across settings or individual backgrounds. However, the analysis did not identify a sufficiently consistent pattern to support systematic comparison by healthcare facility, nationality, or length of nursing experience. Differences were more evident in how individual nurses approached family communication and advocacy than in clear institutional or demographic patterns. Some nurses described a relatively limited role focused on clarifying information, whereas others described becoming more actively involved when misunderstandings or disagreements emerged between families and clinicians.

Overall, these accounts support the interpretation of nurses as intermediaries between families and the clinical team: they could clarify, translate, advocate, and bring family concerns forward, within the boundaries described above.

## 4. Discussion

This study explored ICU nurses’ experiences of supporting families during end-of-life care in Saudi Arabia. Its contribution is not the finding that this work is emotionally demanding and constrained by staffing, which is well established internationally. It is the characterization of a specific position that these nurses occupied and described: an intermediary standing between family and team, holding no formal decision authority while contributing to what a family understood and to what the team heard about that family.

That distinction matters analytically and practically. Much of the literature on nurses in end-of-life care describes advocacy as a role attribute. Our participants described it as a structural position with a defined limit: they could shape the information environment in which a decision was made without any part in making it. Framing nurses’ contribution as influence over understanding rather than participation in decisions is both a more accurate description of what these participants reported and a more defensible basis for intervention, because it identifies what can be strengthened, namely what reaches the team and how, without implying a redistribution of clinical or regulatory authority that the applicable national framework does not provide [12,13]. A contextual consideration, rather than a finding of this study, follows from that framework. Five participants were internationally recruited, and nurses prepared in systems that assign surrogate decision-making authority to families encounter a different arrangement in Saudi Arabia, where a treatment-limitation decision is established by three physicians and families are informed rather than asked to authorize it [12,13]. Adjusting to that arrangement plausibly affects how a nurse explains a decision to a family and how they describe their own role within it; related work on internationally recruited charge nurses in Saudi Arabia has described the cultural adjustment involved in end-of-life practice [18]. Our interview guide did not ask participants to compare regulatory frameworks and we did not analyze accounts by country of preparation; so, this is identified as a consideration warranting attention in future research rather than as a result of the present study.

Participants described a persistent tension between technical responsibilities and relational presence, consistent with literature identifying workload, staffing, and organizational priorities as constraints on family-centered end-of-life care [6,7]. Burnout related to workload and staffing pressures has been documented among ICU nurses in Riyadh [30]. Within this setting, participants’ accounts indicate that recognizing family support as core ICU nursing work, rather than as an additional task undertaken when workload permits, would more accurately reflect the work they described. This is a context-specific proposition arising from these accounts rather than a generalizable mandate, which a study of this design cannot establish.

The grief and self-questioning participants described resemble the emotional strain reported among ICU and palliative care nurses more broadly [6,20], including moral distress [31] and compassion fatigue among critical care nurses in Saudi Arabia [32,33] and internationally [34]. We note two cautions. First, this study measured none of these constructs, and our use of these citations is comparative rather than diagnostic. Second, our own prior work in Saudi palliative and end-of-life settings [20] reported thematically adjacent findings on emotional burden and organizational support.

Organizational conditions were described as central to participants’ experiences. Staffing limitations, limited formal preparation, restricted access to palliative care, and variation in physician collaboration constrained what nurses could provide for families. In contrast, physician recognition of nurses’ bedside observations was described as an enabling condition. These findings suggest that nurses’ capacity to provide family support was shaped not only by their individual skills and commitment but also by the organizational conditions within which end-of-life care was delivered.

Palliative care services in Saudi Arabia have expanded under national health-system reforms [35]. However, the availability of specialist support may still vary across clinical settings. Previous research on structured interventions addressing family needs in the ICU has also reported variable effects across settings, suggesting that organizational context may influence how such interventions are experienced and implemented [36,37]. Similarly, structured communication training, including approaches for discussing difficult news, has been associated with improved nurses’ confidence and performance in challenging conversations [38]. Together, these findings support the importance of organizational preparation and support rather than placing responsibility for effective family communication solely on individual nurses.

Nurses also described the importance of responding to families as individuals rather than assuming that all families had the same needs, beliefs, or preferences. Where supported by the participants’ accounts, this included attention to spiritual needs, family presence, and preferences regarding communication. Nurses’ capacity to provide spiritual care in the ICU may be influenced by workload, preparation, and the availability of institutional support [39]. The present findings should therefore not be generalized to all Saudi, Muslim, or expatriate families. Rather, they indicate the importance of asking individual families about their values, beliefs, spiritual needs, and preferences and adapting support accordingly.

### 4.1. Implications for Practice

Recommendations are labeled by origin so that participant-generated proposals are distinguishable from those inferred by the research team and from those supported by external evidence.

Proposed by participants: Protected time for family communication during active dying; access to a palliative care service at every site; routine opportunity to contribute bedside observations before family meetings; peer forums for talking through difficult deaths.

Inferred by the research team from participants’ accounts: Formal recognition of family support in nursing workload models; structured handover of family concerns to the medical team; induction preparation in end-of-life communication for nurses new to the ICU.

Supported by external evidence rather than by this dataset: Structured communication skills training [38]; structured interventions addressing family needs [36]. This study cannot establish the effectiveness of any intervention, and no such claim is made.

### 4.2. Strengths and Limitations

This study’s strengths include recruitment across three institutions with differing organizational arrangements for adult intensive care and palliative care, coding in the language of data collection rather than in translation, and an explicitly stated analytic position that separates descriptive reporting from interpretive theme development.

This study involved 15 nurses from three urban healthcare facilities in Riyadh. The findings are contextually situated and intended to support transferability rather than statistical generalization. Experiences may differ in rural settings, smaller hospitals, or healthcare systems with different ICU and palliative care structures.

Recruitment through ICU nurse managers may have introduced selection bias. Nurses invited by managers and nurses subsequently referred by participants may have been less willing to describe organizational conditions critically. Although no nurse was excluded on grounds of distress, recruitment was nevertheless initiated through unit managers, and nurses experiencing acute grief at the time of recruitment may have been less likely to volunteer; the most acute emotional strain may therefore be under-represented. This possibility is particularly relevant to Theme 1, which depends substantially on participants’ accounts of staffing, preparation, palliative care access, and interprofessional working conditions. Social desirability is also relevant to Theme 3 because advocacy, emotional presence, and family-centered responsiveness are professionally valued practices. Participants discussing their own conduct with nurse academics drawn from the same national nursing community, though from an organization separate from their employer (Section 2.2), may therefore have presented it favorably. We did not corroborate participants’ accounts through direct observation or through the perspectives of family members, physicians, or other healthcare professionals.

The findings were based on retrospective self-report and may have been influenced by recall. Enabling conditions within Theme 1 were described by fewer participants and were less extensively developed than constraining conditions. All 15 participants are represented by at least one extract in this report. Most interviews were conducted in Arabic (11 of 15) and most participants had Arabic as a first language (11 of 15). Culturally and linguistically situated meanings may therefore be more prominent in, and shaped by, the Arabic-language portion of the dataset, and translation may have affected linguistic nuance despite the procedures described in Section 2.5.2. This study did not include patients, family members, physicians, or other healthcare professionals. Future research should incorporate these perspectives to examine how nurses’ accounts of family support correspond with the experiences of those receiving and participating in care.

## 5. Conclusions

ICU nurses in this study described supporting families during end-of-life care as work performed from an intermediary position: without authority over treatment-limitation decisions, but contributing to what families understood and to what the clinical team heard. This positioning was enabled or narrowed by staffing, formal preparation, palliative care access, and whether physicians treated nurses’ bedside observations as clinically relevant.

Because the constraints participants described were organizational rather than personal, these conditions represent a proximal and potentially modifiable target for intervention, and responsibility for the quality of family support should not rest primarily on individual nurses’ resilience. This study did not compare organizational and resilience-focused approaches, and no such comparison is implied. Whether improving the flow of family information to the clinical team changes decisions, family experience, or quality of dying is a question this study cannot answer and that future research should address.

## Figures and Tables

**Table 1:** Characteristics of the three participating facilities.

Characteristic	Site A (Teaching)	Site B (Teaching)	Site C (Private)
Adult ICU beds	32	28	20
Typical nurse-to-patient ratio	1:1–1:2 day; 1:1–1:2 night	1:1–1:2 day; 1:2 night	1:1–1:2 day; 1:2 night
Specialist palliative care service	Available; multidisciplinary team	Available; consultation-based service	Not routinely available
Family visiting policy	Flexible visiting during end-of-life care; otherwise designated visiting hours	Designated visiting hours; additional access during end-of-life care	Designated visiting hours; additional access subject to ICU approval
Clinical ethics consultation	Available through hospital ethics service	Available through hospital ethics service	Available on referral
Spiritual care service	Available on request	Available on request	Available on request
Written EOL or treatment-limitation procedure	Yes	Yes	Yes
Participants recruited, *n*	6	5	4

EOL, end-of-life; ICU, intensive care unit.

**Table 2:** Recruitment and participant selection process.

Recruitment Stage	Number of Nurses
Nurses approached	21
Declined participation (work-schedule constraints)	4
Interested but no mutually suitable interview time	2
Included in final sample	15

Participants were recruited through nurse-manager distribution, staff-area announcements, and snowball referral. Nurse managers facilitated distribution of recruitment information but were not involved in participant selection, informed consent, interviewing, or access to participant responses.

**Table 3:** Participant characteristics (*n* = 15).

Characteristic	*n* (%) or Range; Mean (SD)
Age, years	28–58; 41.0 (8.2)
Female	12 (80.0)
Male	3 (20.0)
Diploma in Nursing	4 (26.7)
Bachelor of Science in Nursing	8 (53.3)
Master’s degree	3 (20.0)
Total nursing experience, years	2–32; 13.2 (8.1)
ICU experience, years	2–25; 10.4 (6.7)
Experience supporting families during end-of-life care, years	2–20; 8.1 (5.4)
General medical-surgical ICU	7 (46.7)
Cardiac ICU	4 (26.7)
Trauma or neurocritical ICU	4 (26.7)
Site A (teaching hospital)	6 (40.0)
Site B (teaching hospital)	5 (33.3)
Site C (specialized private center)	4 (26.7)
Saudi national	10 (66.7)
Internationally recruited	5 (33.3)
First language Arabic	11 (73.3)
First language other than Arabic	4 (26.7)
Previous formal end-of-life care education ^a^	2 (13.3)
No previous formal end-of-life care education ^a^	13 (86.7)

ICU, intensive care unit. ^a^: Formal end-of-life care education was defined as structured educational preparation specifically addressing end-of-life care, including topics such as communication with patients and families, goals-of-care discussions, symptom management, withdrawal or withholding of life-sustaining treatment, grief and bereavement support, or related palliative care principles. Informal learning acquired through clinical experience, observation of colleagues, or routine workplace exposure was not classified as formal education.

## Data Availability

The qualitative transcripts are not publicly available because they contain potentially sensitive information and are subject to participant confidentiality and ethical restrictions. De-identified data extracts and the coding framework may be made available by the corresponding author. Release requires approval from the King Saud University Institutional Review Board and a signed data-use agreement restricting use to the stated purpose and prohibiting redistribution or any attempt at re-identification.

## Supplementary Materials

The following supporting information can be downloaded at: https://www.mdpi.com/article/10.3390/healthcare14182931/s1, File S1: Interview Guide; File S2: COREQ 32-Item Checklist.

## Abbreviations

The following abbreviations are used in this manuscript:

COREQ	Consolidated Criteria for Reporting Qualitative Research
EOL	end-of-life
ICU	intensive care unit
