# Supplementary material for: Supporting Families During End-of-Life Care in Intensive Care Units: A Qualitative Descriptive Study of Nurses’ Experiences in Saudi Arabia
Source: Healthcare (Basel). 2026 Sep 9;14(18):2931. doi: 10.3390/healthcare14182931 (PMC13606399; doi:10.3390/healthcare14182931)
Supplement: Supplementary file 1 [file healthcare-14-02931-s001.zip › healthcare-4535248-supplementary.pdf]

Supplementary Materials

This file contains File S1 (Interview Guide) and File S2 (completed COREQ 32-item checklist with section references).

File S1. Interview Guide

The semi-structured guide was developed following a review of literature on family support in critical care and end-of-life communication, and consultation with clinical experts in ICU nursing and end-of-life care.

Opening

Participants were reminded that the interview was for research purposes only and was not a clinical assessment, performance evaluation or employment-related activity; that they could decline any question; and that they could pause or stop at any time without giving a reason.

- Could you start by telling me a little about your work in the ICU, and how long you have worked here?
- What kinds of situations involving families at the end of a patient's life do you deal with most often?

Domain 1. Experiences of supporting families during end-of-life care

- Can you describe a recent situation in which you supported a family while a patient was dying or when treatment was being limited?
- What did you actually do for that family?
- What was most difficult about it?
- What, if anything, felt like it went well?

Domain 2. Communication strategies

- How do you explain what is happening to a family when the patient's condition is deteriorating?
- How do you know whether a family has understood?
- How do you handle it when a family asks you something you cannot answer, or that is not yours to answer?
- What happens when the family understands the situation differently from the medical team?

Domain 3. The nurse's role in relation to treatment-limitation and goals-of-care discussions

- What is your role when decisions about resuscitation or limiting treatment are being discussed?
- Are you usually present for those conversations? What do you do during them?
- What happens to a family's concerns after they have told you about them?
- Where do you feel the limits of your role are?

Domain 4. Emotional responses

- How does this part of your work affect you?
- Is there a situation that has stayed with you? What was it about that one?
- What do you do with those feelings, during the shift and afterwards?
- Who do you talk to, if anyone?

Domain 5. Interactions with families and other healthcare professionals

- How would you describe working with the medical team around end-of-life care?
- What happens to what you observe at the bedside? Does it reach the team?
- Can you describe a time when your observations changed something, or when they did not?

- How do families' expectations of you differ from what you are able to give them?

Domain 6. Institutional influences

- What in your unit makes it easier to support families at these times?
- What makes it harder?
- Tell me about staffing and workload during a shift when a patient is dying.
- What preparation or training have you had for these conversations?
- What support is available to you in the hospital: palliative care, spiritual care, ethics consultation?
- How do you handle families' cultural, religious and spiritual needs within the unit's rules?

Domain 7. Recommendations for improvement

- If you could change one thing to make family support easier in your unit, what would it be?
- What would have helped you most when you were new to this work?

Closing

- Is there anything important about this that I have not asked you about?
- Is there anything you would like to add, or anything you would like me to leave out?

Probes used throughout

Open-ended follow-up probes were used to encourage detailed and contextualized accounts, including: “Can you tell me more about that?”; “What happened then?”; “What was that like for you?”; “Can you give me an example?”; “What did you mean by that?”

File S2. COREQ 32-Item Checklist

Consolidated Criteria for Reporting Qualitative Research (Tong, Sainsbury and Craig, 2007; reference 27 in the manuscript). Every item is reported in the manuscript at the section indicated. Where an item was not applicable or was deliberately not claimed, this is stated rather than left blank.

Domain 1: Research team and reflexivity.

No.	COREQ item	Where reported	How it is addressed
1	Interviewer/facilitator	Section 2.5	W.M.A. conducted eight interviews (53.3%); T.E. conducted seven (46.7%). Both are co-authors.
2	Credentials	Section 2.5	Both interviewers hold PhDs in Nursing.
3	Occupation	Sections 2.5, 2.7	Nurse researchers with faculty appointments, College of Nursing, King Saud University.
4	Gender	Section 2.5	W.M.A. male; T.E. female. The wider research team includes male and female researchers.
5	Experience and training	Sections 2.5, 2.7	Clinical backgrounds in critical care nursing; formal training and prior experience in qualitative research.
6	Relationship established	Section 2.5	No relationship before the study. Neither interviewer had a clinical, supervisory, educational or employment relationship with the participating ICUs or any participant.
7	Participant knowledge of the interviewer	Section 2.5	Participants were told before the interview that the interviewer was a nurse researcher conducting the study to understand nurses' experiences of supporting families during ICU end-of-life care.
8	Interviewer characteristics	Section 2.7	Assumptions, professional positioning and their potential influence on interpretation are stated, including the tendency to read difficulty as a communication or organizational deficit.

Domain 2: Study design.

No.	COREQ item	Where reported	How it is addressed
9	Methodological orientation and theory	Section 2.2	Qualitative description with reflexive thematic analysis; contextualist position; primarily semantic coding with selective latent interpretation; experiential orientation; hybrid inductive-deductive coding.
10	Sampling	Section 2.4	Purposive sampling, with eligibility criteria stated in full.
11	Method of approach	Section 2.4; Table 2	Three routes: nurse-manager distribution (n = 5), staff-area announcements (n = 10), snowball referral (n = 6). Participants contacted the research team directly.
12	Sample size	Sections 2.4, Sample Adequacy; Table 3	15 participants. Adequacy justified by information power; saturation not claimed.
13	Non-participation	Section 2.4; Table 2	21 approached; 4 declined (work-schedule constraints); 2 did not proceed because no mutually suitable interview time could be arranged.
14	Setting of data collection	Section 2.5	Private locations selected by participants, typically quiet hospital rooms or offices.
15	Presence of non-participants	Section 2.5	No third party was present at any interview.
16	Description of the sample	Section 3.1; Tables 1 and 3	Age, sex, education, total and ICU experience, experience supporting

			families, ICU subspecialty, site, nationality, first language and prior end-of-life education; institutional characteristics of all three sites.
17	Interview guide	Section 2.5.1; File S1	Guide developed from literature and expert consultation; pilot-tested with three eligible nurses not included in the final sample; provided in full as File S1.
18	Repeat interviews	Section 2.5	No repeat interviews were conducted.
19	Audio/visual recording	Section 2.5	All interviews were audio-recorded with participants' permission.
20	Field notes	Section 2.5	Brief contextual field notes were made immediately after each interview and used to support interpretation.
21	Duration	Section 2.5	35 to 50 minutes; mean 42 minutes (SD 4.6).
22	Data saturation	Sections Sample Adequacy, 2.7	Not claimed. Saturation was explicitly rejected as a stopping rule as inconsistent with reflexive thematic analysis; adequacy was assessed by information power, reviewed after every third interview (n = 3, 6, 9, 12, 15).
23	Transcripts returned	Section 2.5	Transcripts were not returned to participants for correction or comment.

Domain 3: Analysis and findings.

No.	COREQ item	Where reported	How it is addressed
24	Number of data coders	Section 2.6	Two. The first author (W.M.A.) coded all 15 transcripts; a second researcher (M.A.) independently coded six (40%).
25	Description of the coding tree	Section 2.6	A flexible coding framework was used rather than a fixed a priori codebook; codes were revised, merged, expanded, renamed or discarded as analysis progressed.
26	Derivation of themes	Section 2.6	Themes were constructed from the data through the six-phase reflexive process, not identified in advance. Theme 3's organizing concept was developed at the latent level; this is stated in Sections 2.2 and 3.5.
27	Software	Section 2.6	NVivo 12 (QSR International, Melbourne, Australia).
28	Participant checking	Section 2.7	Five participants purposively selected for variation received a two-page summary and two questions; four responded. Treated as interpretive resonance, not validation. Feedback led to strengthened representation of emotional demands.
29	Quotations presented	Sections 3.3 to 3.5	31 extracts, each identified by participant number. All 15 participants are represented; no participant contributes more than three extracts.
30	Data and findings consistent	Sections 3.3 to 3.5	Each analytic claim is accompanied by supporting extracts; divergent accounts are reported in Sections 2.6, 2.7, 3.3.3 and 3.5.5 rather than omitted.
31	Clarity of major themes	Sections 3.2 to 3.5; Figure 1	Three themes, each with a stated organizing concept; relationships shown in the thematic map.
32	Clarity of minor themes	Sections 3.3 to 3.5	Eleven subthemes (3 + 3 + 5), each separately headed. Variation and divergence reported in Section 3.5.5.

Note on item 22: Data saturation is not claimed. Reflexive thematic analysis treats themes as constructed rather than as finite entities awaiting discovery, so saturation is not an appropriate stopping rule. Sample adequacy was assessed by information power instead. This is reported in Sections Sample Adequacy and 2.7 of the manuscript.
